# Dietary supplement mislabelling: case study on selected slimming products by developing a green isocratic HPLC method for their quality control

**DOI:** 10.1038/s41598-022-24830-1

**Published:** 2022-12-24

**Authors:** Noha F. El Azab, Sarah H. Abdelaal, Said A. Hassan, Amira M. El-Kosasy

**Affiliations:** 1 Pharmaceutical Analytical Chemistry Department, Faculty of Pharmacy, Ain Shams University, Cairo, 11566 Egypt; 2 Analytical Chemistry Department, Faculty of Pharmacy, Cairo University, Kasr El-Aini Street, Cairo, 11562 Egypt

**Keywords:** Analytical chemistry, Chemistry

## Abstract

Nowadays, a huge population consumes Dietary supplements for losing weight. Products are often claimed as botanical blends, yet they aren't necessarily safe. Misleading labels are also very common. Thus, validated analytical methods for a wide range of slimming compounds are highly needed. Herein, we present a simple HPLC/PDA method for the quantitation of seven popular slimming ingredients. Studied compounds were Caffeine, Raspberry Ketone, trans-Resveratrol, p-Synephrine, p-Octopamine, p-Hordenine and 2-phenethylamine. After optimization, separation was carried out on a C18 column and mobile phase was a mixture of Acetonitrile:Water containing 0.1% phosphoric acid (50:50, %v/v). The last compound was eluted at 9.76 min. Separation was efficient showing baseline- separated symmetric peaks, without using any gradient programs, organic mobile phase modifiers or modified stationary phases. Method validation was done following ICH guidelines. Calibration curves were linear over wide concentration ranges and calculated LOD values were in the range 0.02–0.09 µg/mL. Method greenness was assessed using Analytical Eco-scale, GAPI and AGREE metric tools. Further, four random sample products purchased from online supplement stores were assayed. Results proved some mislabeling actions. To support our findings, standard addition was carried out and average % recoveries were 96.67 – 101.44% with standard deviation ≤ 2.83 between measurements.

## Introduction

Weight loss Dietary supplements (DS) are now trending over the internet. They are deemed a safe easy way to maintain health and achieve a nice physical appearance. Consumers falsely believe that these products contain natural ingredients and that they have been well tested for safety and efficacy prior to release to the market. Unfortunately, these non-prescription products are ineffective and at times harmful. As per the Dietary Supplement Health and Education Act (DSHEA) in 1994, DS aren't subjected to pre-market approval regarding purity, labeling, safety and efficacy. The FDA reacts only if serious adverse events or new scientific findings were reported post-market release. Consequently, mislabeling fraud is very common. Ingredients are often listed as proprietary blends. Moreover, these multi-ingredient formulas either contain prohibited ingredients or don’t contain the actual active ingredients listed on the label. The consumer should obtain a properly labeled product with a well-known composition. Thus, proper official regulations are mandatory. The National Institute of Health/ Office of Dietary Supplements (NIS/ODS) conducts some regulatory initiatives and encourages the development of standard reference materials for DS ingredients and validated analytical methods to pave the way for reliable quality control practices^1–3^.

As per our web search and in local health shops, slimming formulations currently available to the public contain a wide range of ingredients, either extracted from their natural sources or their synthetic analogues. Among the most commonly used compounds in these OTC products are trans-Resveratrol, Raspberry Ketone and the phenethylamine compounds; 2-Phenethylamine, p-Synephrine, p-Octopamine and p-Hordenine. It was also noticed that high Caffeine content is a major aspect in most weight loss DS.

Resveratrol is a polyphenol that occurs naturally in the skin of red grapes in defense to stress conditions such as fungal infections or environmental distress. It has been widely known for its antioxidant, anti-inflammatory and cardioprotective properties. It is also used as an anti-obesity agent, being an anti-adipocyte that suppresses adipogenesis inducing regulators. Resveratrol exists in both trans- and cis- isomers. However, the trans- form is more sterically stable, abundant and biologically active^4^. As per our literature review, trans-Resveratrol was assayed in supplement products using several techniques including reversed phase high performance liquid chromatography with UV and fluorescence detection^5–8^, fused core column chromatography with UV detection^9^, high performance thin layer chromatography^10^, capillary electrophoresis with UV detection^11^ and electrochemical detection^12^, constant wavelength synchronous spectrofluorimetry^7^and square wave^13,14^ and differential pulse voltammetry^15,16^.

Raspberry ketone is the major aromatic compound that gives the characteristic flavor of raspberry fruits. Among its wide health benefits, it is a popular anti-obesity agent. It induces lipolysis and inhibits intestinal fat absorption^17^. Natural Raspberry Ketone held GRAS (Generally Recognized as Safe) status since 1965^18^. Although it is an abundant ingredient in weight loss DS, the adverse effects of its prolonged consumption aren't yet fully characterized. Few studies reported intravascular hemolysis, hyperglycemia and dose related mortality in mice^19,20^. Various methods have been published for Raspberry Ketone assay in DS products including high performance liquid chromatographic methods with Diode array detection using phenyl columns^21^ and UV detection^22^. It was also determined using high performance thin layer chromatographic^22^, IR-spectroscopic^23^ and spectrofluorimetric^24–26^ methods.

Phenethylamines (PEAs) are sympathomimetics found as appetite suppressants, thermogenic and lipolytic agents in weight loss formulas. 2-Phenethylamine is the most abundant, followed by the Citrus aurantium ingredients; p-Synephrine, p-Octopamine and p-Hordenine. Despite being natural, the high amount of PEAs in DS is possibly of synthetic origin. They are structurally similar to epinephrine and norepinephrine and affect the body's natural neurotransmitter system. Thus, their safety remains questioned. These adrenergic stimulants exert serious cardiovascular adverse events that are exaggerated with caffeine, especially that obese are already at high risk. The World Anti-Doping Agency (WADA) listed 2- Phenethylamine and p-Octopamine as prohibited substances and included p-Synephrine, p-Hordenine and Caffeine in the 2021 monitoring list to monitor the pattern of their abuse among athletes. Yet, they are still seen to the public^2,27^. To our knowledge, analytical methods have been published for either qualitative screening or quantitation of PEAs. These include monolithic column chromatography with acidic potassium permanganate chemiluminescence^28^, reversed phase high performance liquid chromatography with UV detection^29–37^, fluorescence detection^29,32,38^ and mass detection^33,35,39–41^, gas chromatography with mass detection^42^, capillary electrophoresis^43^, square wave and differential pulse voltammetry^44,45^ and NMR spectroscopy^46^.

To date, no general method is available in literature for the simultaneous assay of the previously mentioned weight loss ingredients. Only several methods have been published for the separation of mixtures of p-Synephrine, p-Octopamine and p-Hordenine^32,38,40^and at times with Caffeine^31,36^. Proper product labeling requires reliable validated analytical methods and a universal method is still indispensable.

Herein, we aimed to propose a versatile method that could be feasibly applied for quality control practices in regulatory laboratories. We developed a validated reversed phase high performance liquid chromatographic method for the simultaneous determination of Caffeine, trans-Resveratrol, Raspberry Ketone, p-Octopamine, p-Synephrine, p-Hordenine and 2-Phenethylamine, employing a photodiode array detector. The separation was achieved isocratically using only acetonitrile and acidified water in a short run time. Formal validation was done in accordance to ICH guidelines and method was applied to the analysis of random DS samples purchased from online health shops.

## Experimental

### Standards and re-agents

Caffeine anhydrous (1, 3, 7-Trimethylpurine-2, 6-dione, CAS no. [58-08-2], certified to be ≥ 99.5%), 2-Phenylethylamine HCl (CAS no. [156-28-5], certified to be ≥ 98.0%), p-Hordenine HCl (4-(2-(Dimethylamino) ethyl) phenol hydrochloride, CAS no. [6027-23-2], certified to be ≥ 99.5%), p-Octopamine HCl (4-(2-amino-1-hydroxyethyl) phenol hydrochloride, CAS no. [770-05-8], certified to be ≥ 99.5%), p-Synephrine HCl (4-[1-Hydroxy-2-(methylamino) ethyl] phenol hydrochloride, CAS no. [5985-28-4], certified to be ≥ 99.5%), trans-Resveratrol (trans-3, 5, 4′-Trihydroxystilbene, CAS no. [501-36-0], certified to be ≥ 99.0%) and Raspberry Ketone (4-(4-Hydroxyphenyl)-2-butanone, CAS no. [5471-51-2], certified to be ≥ 99.5%) were purchased from China (Baoji Guokang Biotechnology Co. Ltd); Acetonitrile, water (Fisher Chemical, UK) and 85% ortho-phosphoric acid (Merck, Darmstadt, Germany) were HPLC reagent grade.

### Instrumentation and chromatographic conditions

Analysis was carried out using HPLC Waters Alliance e2695 separating module (Waters Co., MA, USA) equipped with a quaternary solvent system, in-line vacuum degasser, 100 µl injection loop; autosampler, heated column compartment and photo-diode array (PDA) detector. Analytical column was RP Spherisorb® ODS-2; 250 × 4.6 mm with 5 µm particle size (Waters Co., MA, USA). Empower-3 chromatography data software was used for data collection and acquisition. Column compartment and sample tray were kept at 25 °C. Mobile phase was a mixture of Acetonitrile: 0.1% phosphoric acid in ratio 50:50%v/v. 20 µl of samples were injected in triplicates and isocratically eluted with flow rate 0.9 mL/min. Total run time was 11 min and column was equilibrated with mobile phase for 5 min between single injections. PDA detection was carried out at 205 nm for Caffeine and 2-Phenylethylamine, 225 nm for Raspberry Ketone, p-Octopamine, p-Synephrine and p-Hordenine and 305 nm for trans-Resveratrol. Separation efficiency was verified by system suitability results.

### Supplement products

Four different dietary supplement samples indicated for weight loss were purchased from online health shops. Sample (1) was labeled to contain a 280 mg proprietary blend of Caffeine anhydrous, Synephrine HCl and Octopamine HCl; per tablet. Sample (2) was claimed to contain 125 mg 2-Phenylethylamine HCl, 80 mg Caffeine anhydrous, 15 mg Hordenine HCl and 1 mg Synephrine HCl; per tablet. Sample (3) was claimed to be a mixture of 400 mg of Raspberry Ketone (4%), 400 mg Caffeine and Grape extract equivalent to 200 mg; per capsules serving. Sample (4) was labeled to contain Raspberry Ketone powder 300 mg, Green tea leaf extract 200 mg, Caffeine anhydrous 100 mg and a proprietary blend of Apple cider vinegar powder, Grapefruit powder, Kelp powder, Acai fruit powder, African mango seed extract and 10% Resveratrol extract; per capsules serving. All samples were analysed before their expiration date.

### Standard solutions

Separate stock solutions (100 µg/mL) of all drugs were prepared using water, except for trans-Resveratrol, which was dissolved in acetonitrile: water (1: 9, %v/v). All stock solutions were stable for 7 days when stored at 4 °C. Working standard solutions for construction of calibration curves were freshly prepared by appropriate linear aliquot dilution of stock solutions in mobile phase.

### Sample preparation

Emptied capsule contents or finely powdered tablets equivalent to one serving size were accurately weighted, dissolved in water (samples 1 and 2) or acetonitrile: water (1: 9, %v/v) (samples 3 and 4), sonicated for 10 min, filtered and stock solutions of each sample were prepared. Appropriate dilutions of each sample were prepared in mobile phase to fall within the calibration range and analysed under the previously mentioned chromatographic separation conditions. Extraction efficiency was further evaluated using standard addition technique. Known amounts of pure standards were added to commercial samples; those mixtures were then subjected to the entire sample preparation and quantitation steps. Recoveries were calculated by subtracting the concentration of the non-spiked samples from the total concentration of the spiked samples.

## Results and discussion

In view of the poor DS regulation, the existence of analytical methods to simultaneously identify and quantify different constituents in these products is very important to the field of health surveillance. This study sheds the light on seven popular slimming compounds; namely: Caffeine, trans-Resveratrol, Raspberry Ketone, p-Octopamine, p-Synephrine, p-Hordenine and 2-Phenethylamine. A simple, ecological and cheap RP-HPLC/PDA method was developed and validated for their simultaneous determination. To date, analytical methods are published for quantitation of a single compound or combination of few of them. Moreover, these methods employ long gradient programs and propose minimal validation data^5,22,30,33^. Thus, this proposal can be put forward as a general green, simple and valid method for the quality control of a wide variety of commercially available DS products.

### Method optimization

**Figure 1:**
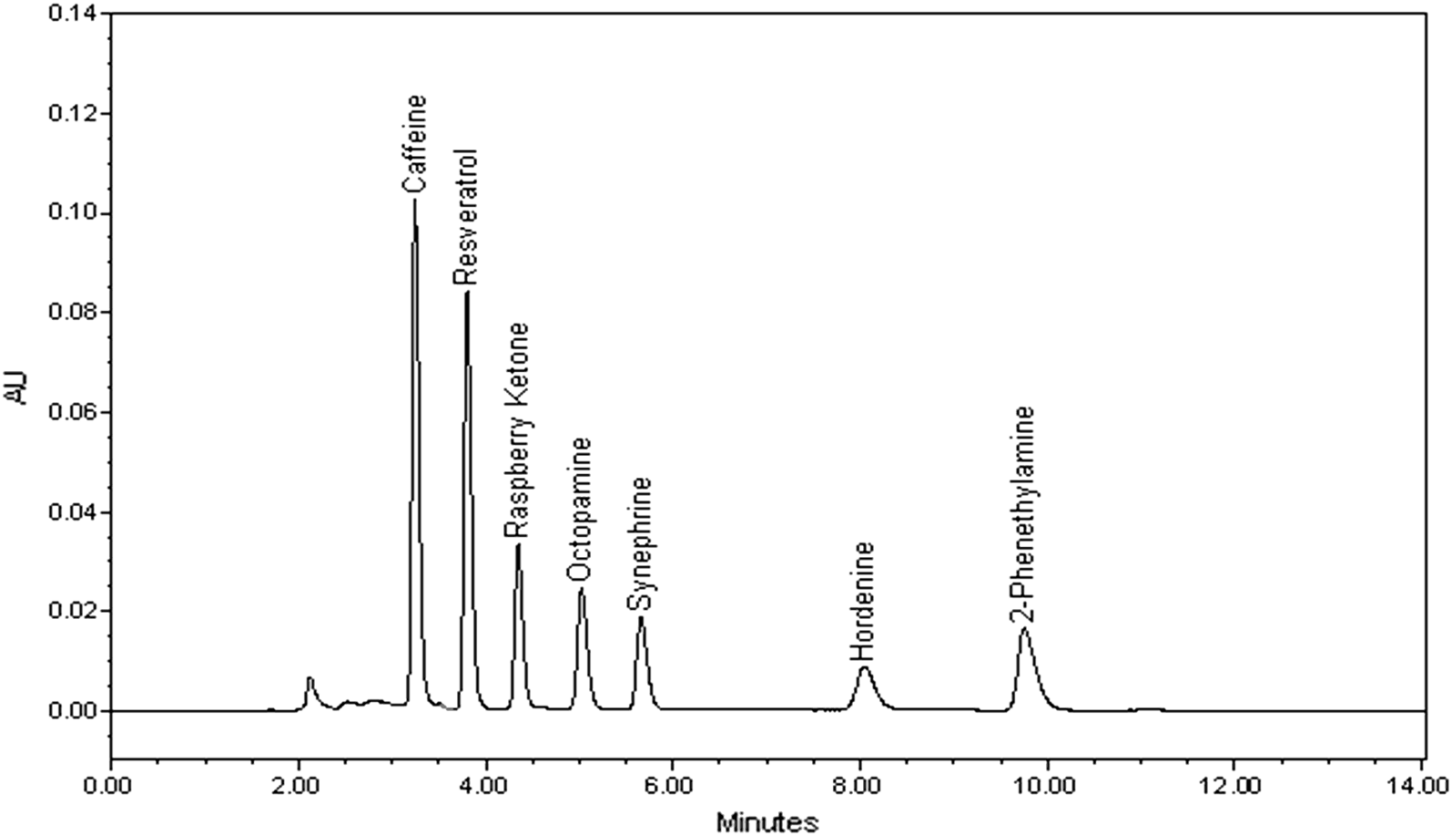
HPLC–PDA chromatogram of standard solution of the studied drugs at 205 nm for caffeine and 2-phenethylamine, 225 nm for Raspberry Ketone, p-Octopamine, p-Synephrine, p-Hordenine and 305 nm for trans-Resveratrol (2 µg/mL each).

On our chemical investigation of all mentioned compounds, we aimed to develop a green method that shows best resolution, peak shape and sensitivity using a conventional ODS column in a very short run time. Chromatogram showing all separated compounds is presented in Fig. 1. A standard C18 column was preferred as it is the most popular among quality control labs. However, all analytes were basic hydrophilic and separation of the basic PEAs on unmodified reversed phase silica was challenging. At neutral water pH, basic analytes are strongly retained on silanol residues and so peaks didn't appear in the relevant chromatograms. At low pH, unionized silanols and ionized basic analytes limit ion-exchange and let compounds be eluted^47^. Thus, methods in literature always used acidic buffers or ion pairing agents (e.g.: heptane sulphonate or sodium lauryl sulphate) through gradient mobile phase composition and/or gradient flow rate programs in long run times^31,48^. These mobile phase additives shorten the column lifetime and tedious gradient procedures aren't feasible for quality control practices. Only two methods were published in literature presenting a low concentration phosphoric acid in mobile phase instead of using organic acid modifiers. Insufficient resolution was observed between Octopamine and Synephrine peaks despite that both used gradient mobile phase composition and/or flow rate gradient elution^36,37^. The proposed method was optimized regarding mobile phase composition, flow rate, detection wavelength and injection volume. Mixtures of methanol: 0.1% phosphoric acid and acetonitrile: 0.1% phosphoric acid were tested in different proportions and flow rates. Acetonitrile was more promising than methanol due to its lower viscosity and UV cutoff. Methanol chromatograms were noisy and this reduced method sensitivity^49^. Acetonitrile: 0.1% phosphoric acid mixture was tested in the ratios, 30:70, 50:50 and 70:30, %v/v. In low acetonitrile ratio, Octopamine and Synephrine peaks co-eluted. Increasing acidified water proportion caused relative increase in protonation of bases and decreased their retention on column. In high acetonitrile ratio, last compound was eluted at 30 min along with co-elution of Caffeine and trans-Resveratrol peaks. The best baseline separation of the seven compounds was observed with isocratic elution of acetonitrile: 0.1% phosphoric acid mixture (50:50, %v/v). Isocratic elution was sufficient to provide satisfactory baseline separation with resolution between peaks more than 2, eliminating the need for the previously published laborious gradient programs and organic modifiers in the mobile phase. Blank solvent samples were injected and no carryover was detected between runs. Different pump flow rates were tested, a flow rate 0.9 mL/min showed the best resolution between peaks along with the least possible run time with the last compound eluted at 9.76 min. Although Raspberry Ketone and PEAs showed nearly similar UV spectra, they were differently eluted from the column under optimized conditions. Each analyte was detected at its λ_max_; 205 nm for Caffeine and 2-Phenethylamine, 225 nm for Raspberry Ketone, p-Synephrine, p-Octopamine and p-Hordenine and 305 nm for trans- Resveratrol. Optimum injection volume was 20 µl, above which peak shapes were distorted. Hence, both detection wavelengths and injection volume attributed to the method sensitivity.

### Method validation

All validation parameters for our proposed method were calculated as per the ICH Q2 (R1) guidelines^50^.

#### Selectivity

**Figure 2:**
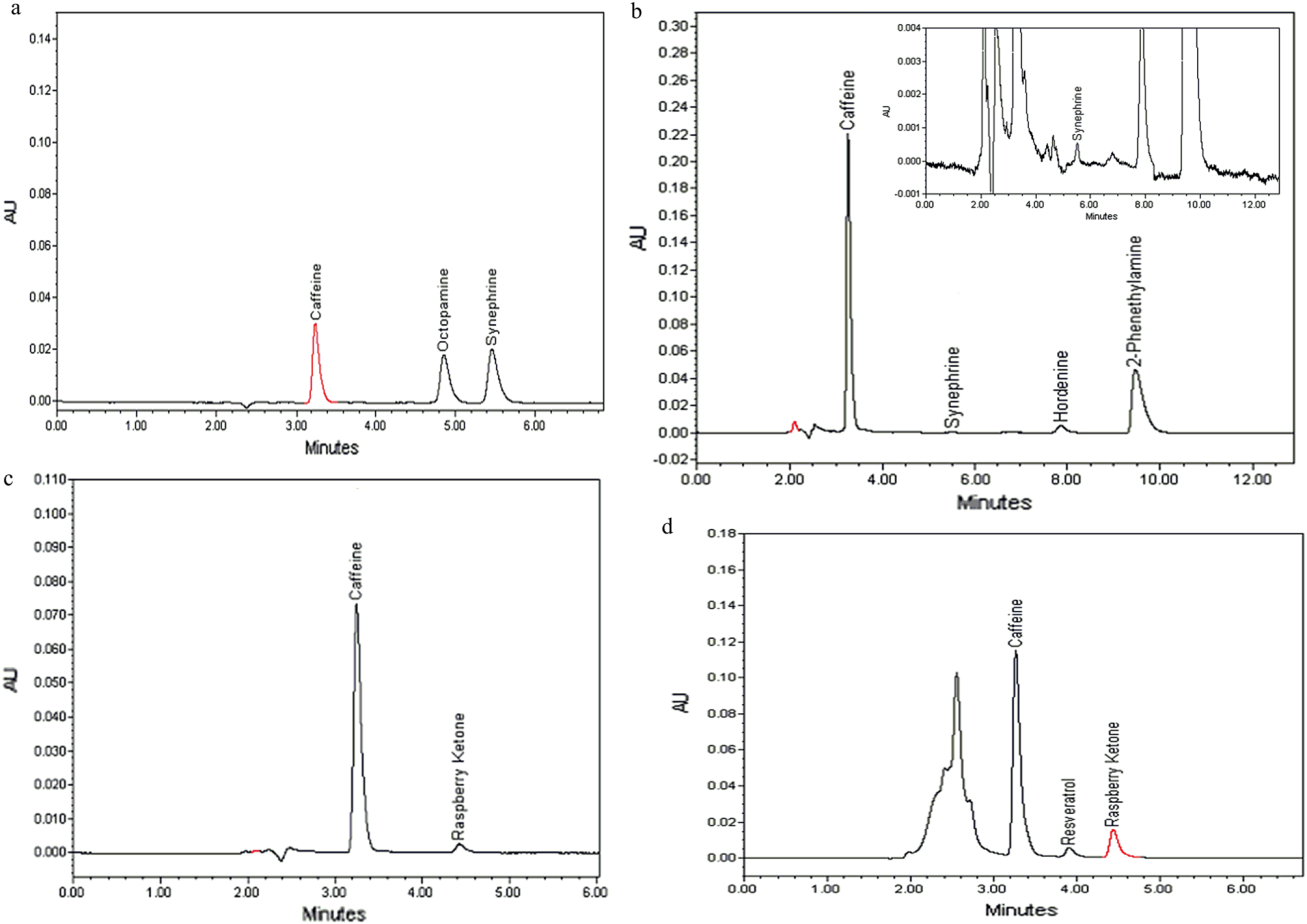
HPLC–PDA chromatogram of Dietary supplement samples (**a** sample 1, **b** sample 2, **c** sample 3, and **d** sample 4).

Under the proposed chromatographic conditions, all target analytes were separated all the way to the baseline, showing well resolved peaks for both pure standard solutions and tested supplement samples. Representative chromatograms (Figs. 1 and 2) show no interfering peaks at the retention times of the studied drugs.

#### Linearity

Five to seven point calibration curves were constructed for the studied drugs, correlating average peak area at the selected detection wavelength to the corresponding concentration and regression equations were computed. All linearity ranges and regression parameters are listed in Table 1. All drugs were linear over acceptably wide concentration ranges with correlation coefficient values higher than 0.999.

**Table 1:** Regression parameters and assay validation results of the proposed HPLC method.

	Caffeine	Resveratrol	Raspberry ketone	Octopamine	Synephrine	Hordenine	2-phenethyl amine
Detection wavelength (nm)	205	305	225	225	225	225	205
Retention time (min.)	3.24	3.8	4.35	5.13	5.66	8.05	9.76
SD of retention time (min.)	0.01	0.07	0.05	0.17	0.1	0.13	0.001
Range (µg/mL)	0.30–10.00	0.10–4.00	0.10–5.00	0.10–5.00	0.10–5.00	0.30–5.00	1.00–15.00
Correlation coefficient (r)	0.9995	0.9994	0.9993	0.9997	0.9996	0.9997	0.9993
Slope	162,959	135,422	62,028	53,923.9	44,116.6	36,927.8	61,881.6
Intercept	28,867.7	11,072	4920.12	1162.77	1639.91	-305.5	11,128.4
LOD (µg/mL)	0.04	0.03	0.02	0.02	0.03	0.09	0.07
LOQ (µg/mL)	0.12	0.09	0.06	0.06	0.09	0.27	0.21
Accuracy (mean recovery % ± SD)	98.80 ± 0.85	100.21 ± 2.18	98.71 ± 1.53	100.28 ± 1.18	100.44 ± 2.32	100.04 ± 2.23	101.06 ± 0.30
Intra-day precision (%RSD)	0.5	0.55	0.83	0.54	0.21	0.8	0.73
Inter-day precision (%RSD)	1.87	0.81	1.72	1.32	0.87	1.55	1.37

#### Limit of detection (LOD) and limit of quantitation (LOQ)

Method sensitivity was assayed in terms of LOD and LOQ values. Determination of LOD and LOQ was based on calibration data using the residual standard deviation of regression line in the range of detection limit (σ) and the slope of the calibration curve (s), applying the formula LOD = 3.3 × σ/s and LOQ = 10 × σ/s. Results are presented in Table 1.

#### Accuracy and precision

Accuracy was evaluated by analyzing different concentrations of pure drug solutions across the linear ranges. Accuracy results were expressed as average percentage recoveries. The percentage relative standard deviation (%RSD) was calculated between a set of triplicate measurements of three different concentrations of each drug performed on the same day and on three consecutive days for expressing both Intra-day and Inter-day precision; respectively. Calculated results, expressed in Table 1, prove that our method is accurate and precise as indicated by percentage recoveries close to 100% and %RSD values less than 2%.

#### System suitability

System suitability testing evaluates the whole components of an analytical system and efficiency of separation. During method optimization, System suitability parameters were calculated using Empower® software as described in FDA guidance for industry on Validation of Chromatographic Methods and USP-NF General Chapter <621> Chromatography–system suitability^51^. Under the selected conditions, results (Table 2) were within the acceptable values verifying that the chromatographic system is efficient showing well resolved symmetric peaks. Thus, the proposed method is adequately valid for its intended purpose.

**Table 2:** System suitability parameters for the assay of the studied drugs using the proposed HPLC method.

Parameter	Caffeine	Resveratrol	Raspberry ketone	Octopamine	Synephrine	Hordenine	2-phenethyl amine	Reference value^59^
Capacity factor (K')	0.53	0.8	1.06	1.45	1.68	2.8	3.61	>2
Injection repeatability (%RSD)^a^	0.38	0.35	0.66	1.19	0.72	1.65	0.17	≤ 1
Tailing factor (T_f_)	1.31	1.26	1.22	1.19	1.2	1.23	1.9	≤ 2
Number of theoretical plates(N)	9475.74	10,582.6	12,527.3	13,001.5	13,530.8	10,002	14,412.2	>2000
Resolution^b^	5.25	3.3	3.67	3.53	9.61	5.42		≥ 2
Selectivity factor (α)	**–**	1.69	1.28	1.26	1.22	1.7	1.3	>1

^a^Peak area of five replicates of standard solution.

^b^USP Resolution calculated between a peak and its preceding peak.

#### Robustness

Robustness testing is a measure of the degree of method reliability to small but deliberate variation in operating conditions. It also highlights and set limits for critical method parameters. Multiple samples at the same concentration level were analyzed after varying pump flow rate (± 0.05 mL/min) and mobile phase composition (± 2%) and %RSD of assay was calculated at each typical variation. Results (Table 3) reveal that method was robust with respect to flow rate changes. However, mobile phase composition is a critical method parameter that affects resolution between peaks. Increasing 0.1% phosphoric acid concentration in mobile phase by 2% didn’t affect %drug assay, but resolution decreased to be 1.74 and 1.81 for Raspberry Ketone-Octopamine and Octopamine-Synephrine peaks; respectively.

**Table 3:** Robustness results of the proposed HPLC method expressed as %RSD of the drug assay.

Condition		Caffeine	resveratrol	Raspberry Ketone	Octopamine	Synephrine	Hordenine	2-phenethyl amine
Flow rate 0.9 mL/min	Flow rate 0.85 mL/min	0.62	1.85	0.42	1.04	1.19	1.77	1.69
	Flow rate 0.95 mL/min	0.56	0.32	1.53	0.52	1.74	0.93	0.99
Acetonitrile: 0.1% phosphoric acid 50:50,%v/v	Acetonitrile: 0.1% phosphoric acid 52:48,%v/v	0.65	0.44	1.14	0.39	1.7	1.34	1.44
	Acetonitrile: 0.1% phosphoric acid 48:52,%v/v	0.2	1.14	0.29	0.35	1.25	0.41	0.56

### Analysis of real samples

**Figure 3:**
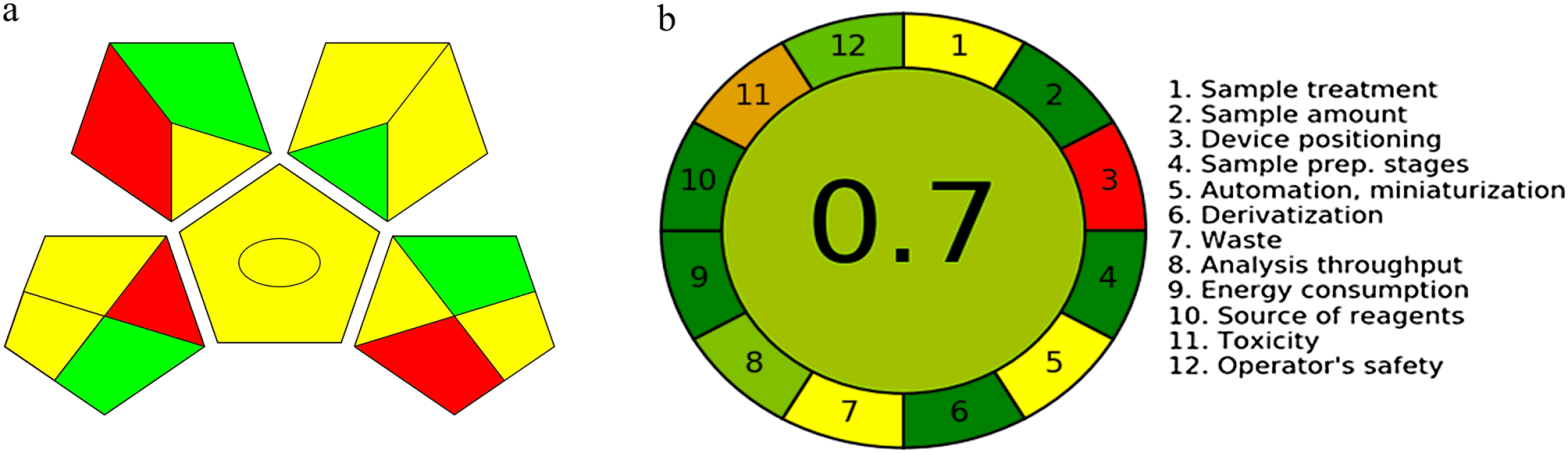
(**a**) GAPI—green analytical procedure index (**b**) AGREE – Analytical GREEnness Calculator assessments of our proposed methods.

After validation, the viability of the proposed method was endorsed. Four different DS samples were purchased from online retailers and analyzed using our mentioned procedures. Extraction was simply done using water, except for samples claimed to contain Resveratrol, that 10% acetonitrile in water was used. Compounds were identified on basis of relevant retention times, UV spectra and spiking samples with known amounts of pure standards for spectrally similar compounds. Representative chromatograms (Fig. 3) showed no interfering peaks in the relevant regions. Moreover, peak purity testing was performed using Empower® PDA software to exclude that possible impurities or degradates were co-eluted with studied compounds. Evaluation involved comparing a purity angle to threshold angle for each peak. Purity angle, the degree of spectral homogeneity, compares spectra across each point in the peak to the spectrum at the peak apex. Threshold angle is the largest possible purity angle attributed to co-elution, noise, solvent and detector error. An impurity is detected within a peak when its purity angle exceeds its threshold angle. In all tested DS products, peaks corresponding to studied drugs showed purity angle lower than threshold angle (Table 4) demonstrating spectral homogeneity, therefore, confirming the purity of eluting peaks^52^.

**Table 4:** Assay and standard addition results of commercial dietary supplement samples using the proposed HPLC method.

	Compound name	Peak purity		Declared amount (mg/tab or Cap)	Found amount (mg/tab or Cap)^c^	% of label claim	Standard addition	
		Purity angle^a^	Threshold angle^b^				(% Recovery^d^)	SD
Sample 1	Caffeine	1.67	3.34	–	115.55	–	99	2.6
	Synephrine	2.88	4.8	**–**	101.76	**–**	101.11	2.71
	Octopamine	3.9	5.8	**–**	65.8	**–**	100.11	2.83
	Total blend			280	283.11	101.11		
Sample 2	2-phenethyl amine	4.57	8.57	125	126.9	101.52	99.89	1.87
	Caffeine	0.96	2.02	80	77.1	96.4	100.16	1.34
	Hordenine	8.79	12.64	15	14.85	99	99.95	2.1
	Synephrine	21.69	46.74	1	0.98	98	100.49	2.82
Sample 3	Raspberry ketone	24.64	41.43	8	3.81	47.63	100	2
	Caffeine	0.36	1.49	200	206.72	103.36	98.78	0.84
	Grape extract (resveratrol)			100	N.D	N.D	96.67	0.58
Sample 4	Raspberry ketone	10.96	38.2	150	4.42	2.95	100.33	2.08
	Caffeine	3.26	6.04	50	48.84	97.68	101.36	1.82
	Resveratrol	13.29	53.13	**–**	0.8	**–**	101.44	1.71

^a^Purity angle compares spectrum of each point in the peak to the peak apex spectrum.

^b^Purity threshold: solvent angle + noise angle.

^c^Average of three determinations.

^d^Average of three determinations on three concentration levels.

–Ingredients were labeled as proprietary blends without stating the exact amount of each.

N.D. not detected below LOD.

Contents in samples were measured and compared relevant to label claims. Then samples were spiked with known amounts of pure standards to further identify compounds and evaluate the extraction efficiency. Assay and standard addition results are presented in Table 4.

For samples (1) and (2), measured amounts were in fairly good agreement with those on the label. Sample (1) contained a total intake of p-Synephrine and p-Octopamine higher than the safe levels, in addition to the high amount of Caffeine^2,53^. This probably explains the manufacturer's intention to mislead the consumer about the active agent content, that the mixture was labeled as a proprietary blend without stating the exact amount of each component. Despite that assay results of sample (2) matched with label claim, it is crucial to alert that it contains a very high level of the prohibited synthetic 2-Phenethylamine and yet is still available for sale to the public. For the other tested samples, results showed that the actual contents largely varied from the label claims. No resveratrol was detected above method LOD in sample (3), although it was labeled to contain 100 mg of grape extract. Moreover, it contained nearly half of its labeled Raspberry Ketone content. Similarly, Sample (4) contained approximately 3% of the labeled Raspberry Ketone, although it was declared as the main active agent in the product. To support the accuracy of our findings, standard addition was applied to the faulty samples and average recoveries varied between 96.67 and 103.33%.

### Green assessment

#### Analytical eco-scale assessment

Method greenness was assessed using the ''Eco-Scale'' tool. In evaluation, it considers hazard and amount of used re-agents, energy use, occupational hazard and waste generation. Each item is given a number of penalty points (PPs) and the total PPs are subtracted from an ideal total score of 100. Eco-scale scores >75, >50 or < 50 describe excellent, acceptable or inadequate greenness; respectively. Eco-scale score, calculated as described by Namiesnik et al.^54^, was 90 proving that our method is an excellent green one (Table 5).

**Table 5:** Penalty points (PPs) of the proposed method according to the analytical eco-scale assessment tool.

Re-agents	Amount	Hazard pictograms	Signal word	PPs
Water	˂ 10 mL	–	–	0
Acetonitrile	˂ 10 mL	Flame, Skull	Danger	4
Ortho-phosphoric acid	˂ 10 mL	Corrosion	Danger	2
Instruments		Energy		PPs
HPLC		≤ 1.5kWh per sample		1
Vacuum pump		˂ 0.1 kWh per sample		0
Sonicator		˂ 0.1 kWh per sample		0
Occupational hazard		Hermetization in analytical process		0
Waste		1–10 mL		3
Total PPs				10
Eco-scale score				90

#### Green analytical procedure index (GAPI)

GAPI is a semi-quantitative tool that evaluates all stages of an analytical procedure from sample collection to preparation to final analysis. It also shows whether a method is qualitative or quantitative. Assessment involves a three colored symbol that consists of five pentagrams. Each section is colored green, yellow or red for high, mid and low environmental friendliness; respectively. This metric visually highlights the variations among different analytical methodologies^55,56^. GAPI assessment of our proposed analytical method was calculated using ComplexGAPI® software^57^ and results are shown in Fig. 3a.

#### Analytical GREEnness metric approach (AGREE)

AGREE is an automated metric that considers the 12 principles of Green Analytical Chemistry (SIGNIFICANCE) during evaluation. Each criterion is given a score from 0 to 1. Final result is a pictogram divided into 12 segments. Each segment is colored green, yellow or red in different color intensities, according to its greenness. The width of each segment corresponds to its weight assigned by user. Finally, a total score of the whole analytical methodology is presented in the middle of the pictogram with a color corresponding to this score, where dark green is associated with values close to 1.This method is considered unique for its flexibility and that it not only considers the hazards of an analytical method but its output as well^58^. AGREE assessment of our proposed analytical method was calculated using AGREE® software and results are shown in Fig. 3b.

## Conclusion

In this manuscript, the problem of DS mislabeling in market was assessed. For the assay of weight loss DS, an isocratic RP-HPLC method with diode array detection was developed for the separation and quantitation of seven popular lipolytic agents in weight loss DS. The proposed method is environment-friendly in which only acetonitrile and acidified water are enrolled for complete separation in an adequately short run time. Separation was efficient showing well resolved Gaussian shaped peaks. Validation was done in accordance to ICH guidelines and results proved that the method is sensitive, accurate, precise and robust across a wide concentration range. Further, it was applied to the assay of four DS products randomly purchased from online stores. Our results revealed the discrepancy between labeled and actual contents in two of the analyzed samples. The other two samples agreed with label claims. However, it's worth alerting that these two samples contained higher than safe levels of caffeine and PEAs that are already on monitoring/Prohibited WADA list and yet still available to the public. Finally, we believe that this simple, green, efficient and cost-effective procedure will contribute to enhancement of DS regulatory practices.

## Data Availability

The datasets generated and analysed during the current study are available from the corresponding author on reasonable request.
