# Author Correction: Dietary supplement mislabelling: case study on selected slimming products by developing a green isocratic HPLC method for their quality control

**DOI:** 10.1038/s41598-023-28976-4

**Published:** 2023-01-31

**Authors:** Noha F. El Azab, Sarah H. Abdelaal, Said A. Hassan, Amira M. El‑Kosasy

**Affiliations:** 1 Pharmaceutical Analytical Chemistry Department, Faculty of Pharmacy, Ain Shams University, Cairo, 11566 Egypt; 2 Analytical Chemistry Department, Faculty of Pharmacy, Cairo University, Kasr El‑Aini Street, Cairo, 11562 Egypt

Correction to: *Scientific Reports* 10.1038/s41598-022-24830-1, published online 24 December 2022

The original version of this Article contained an error in the spelling of the author Noha F. El Azab, which was incorrectly given as Noha F. El-Azab.

The original Article has been corrected.

